# AT_1_ Receptor Induced Alterations in Histone H2A Reveal Novel Insights into GPCR Control of Chromatin Remodeling

**DOI:** 10.1371/journal.pone.0012552

**Published:** 2010-09-03

**Authors:** Rajaganapathi Jagannathan, Suma Kaveti, Russell W. Desnoyer, Belinda Willard, Michael Kinter, Sadashiva S. Karnik

**Affiliations:** 1 Department of Molecular Cardiology, Cleveland Clinic Foundation, Cleveland, Ohio, United States of America; 2 Proteomics Core, Lerner Research Institute, Cleveland Clinic Foundation, Cleveland, Ohio, United States of America; George Mason University, United States of America

## Abstract

Chronic activation of angiotensin II (AngII) type 1 receptor (AT_1_R), a prototypical G protein-coupled receptor (GPCR) induces gene regulatory stress which is responsible for phenotypic modulation of target cells. The AT_1_R-selective drugs reverse the gene regulatory stress in various cardiovascular diseases. However, the molecular mechanisms are not clear. We speculate that activation states of AT_1_R modify the composition of histone isoforms and post-translational modifications (PTM), thereby alter the structure-function dynamics of chromatin. We combined total histone isolation, FPLC separation, and mass spectrometry techniques to analyze histone H2A in HEK293 cells with and without AT_1_R activation. We have identified eight isoforms: H2AA, H2AG, H2AM, H2AO, H2AQ, Q96QV6, H2AC and H2AL. The isoforms, H2AA, H2AC and H2AQ were methylated and H2AC was phosphorylated. The relative abundance of specific H2A isoforms and PTMs were further analyzed in relationship to the activation states of AT_1_R by immunochemical studies. Within 2 hr, the isoforms, H2AA/O exchanged with H2AM. The monomethylated H2AC increased rapidly and the phosphorylated H2AC decreased, thus suggesting that enhanced H2AC methylation is coupled to Ser1p dephosphorylation. We show that H2A125Kme1 promotes interaction with the heterochromatin associated protein, HP1α. These specific changes in H2A are reversed by treatment with the AT_1_R specific inhibitor losartan. Our analysis provides a first step towards an awareness of histone code regulation by GPCRs.

## Introduction

G protein-coupled receptors (GPCR) are the largest family of transmembrane molecules that sense environmental signals in mammalian cells. Accordingly, >50% of the drugs in therapy target GPCRs, suggesting the physiological and pathophysiological significance of GPCRs [1], [2], [3], [4]. GPCRs regulate global gene expression programs leading to diverse integrated responses such as proliferation, differentiation and apoptosis in cells, but the mechanisms by which GPCRs modulate the structure-function relationship of chromatin is unclear. It is well established that GPCR activation mobilizes transacting factors, such as protein kinases/phosphatases (e.g., ERK and calcineurin) histone deacetylases/acetylases (e.g., HDACs and HATs), and transcription factors (e.g., NFAT, NK2, MEF2, GATA, Tbx and STAT), which either ‘induce’ or ‘repress’ gene expression [5], [6], [7], [8], [9]. However, GPCR-modulation of *cis*-acting epigenetic mechanisms which could alter the ‘expressibility’ of genes is unknown. The *cis*-acting epigenetic regulatory mechanisms could cause long-term changes in gene expression and would therefore affect sets of genes in chromatin. The conceptual and operational aspects of this type of regulation by GPCRs are unexplored.

Chromatin is a complex of DNA with histone and non-histone proteins. The fundamental unit of the chromatin structure is the nucleosome, which consists of 146 base pairs of DNA, wrapped around an octamer of the core histones H2A, H2B, H3, and H4. Histones constitute 50% of the mass of chromatin and are subject to a wide range of post-translational modifications (PTM). The amino-terminal tails of each of the four core histones are targets for several types of covalent modifications, including acetylation, methylation, and phosphorylation. In addition, histone isoforms encoded by distinct genes exist and are regulated in response to specific signals [10]. GPCRs likely regulate histone isoforms and PTM to modulate gene expression, but this aspect has not to date been systematically investigated. We propose that histone alterations are an index associated with the activity of GPCRs governing the phenotype of a cell.

**Figure 1:**
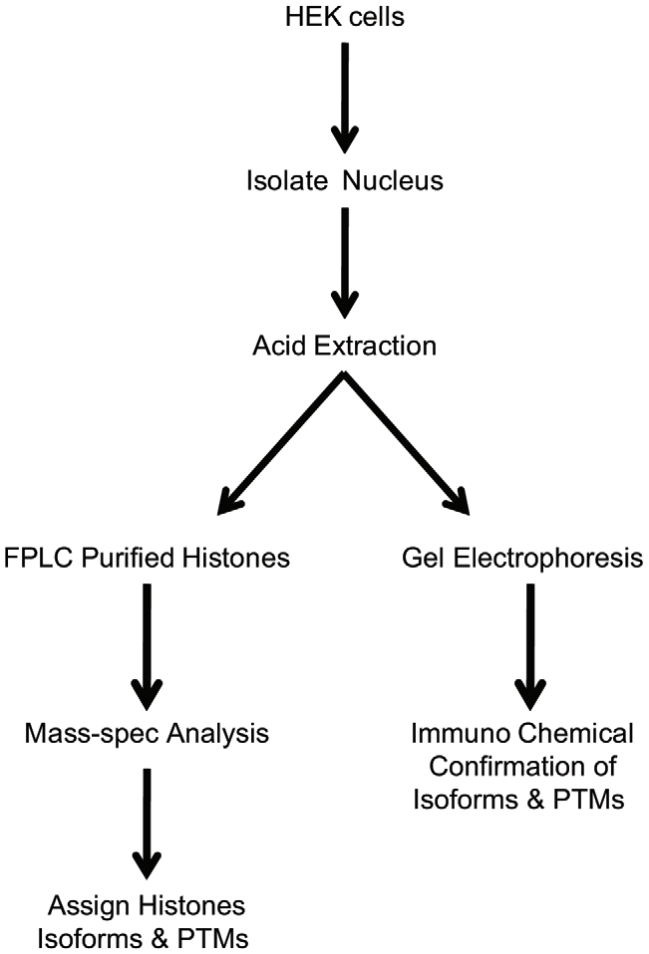
Experimental flow chart.

The angiotensin type 1 receptor (AT_1_R) is an ideal model system for exploring modulation of histone imprints by a GPCR. Angiotensin II is the primary effector hormone of the renin-angiotensin system. This hormone regulates the cardiovascular response through the AT_1_R [11], [12] in diseases such as hypertension, atherosclerosis, heart-failure, and diabetes [13], [14], [15], [16], [17]. Pathological vascular remodeling and cardiac hypertrophy [18], [19], [20], [21] can be treated with AT_1_R blocking (ARB) drugs, thus suggesting that activated AT_1_R contributes to changes in the phenotype of cells *in vivo*. We speculate that a relative abundance of histone PTM and histone isoforms in chromatin is a potential index of the activation states of AT_1_R in cells. Here, we utilize mass spectrometry combined with biochemical analysis to characterize the relative abundance of the histone H2A in a human cell line displaying distinct activation states of the AT_1_R (Fig. 1). We have documented alteration in the levels of specific histone H2A modifications, as well as isoforms when the activation state of the AT_1_R changes.

## Results

### General strategy for histone analysis

**Figure 2:**
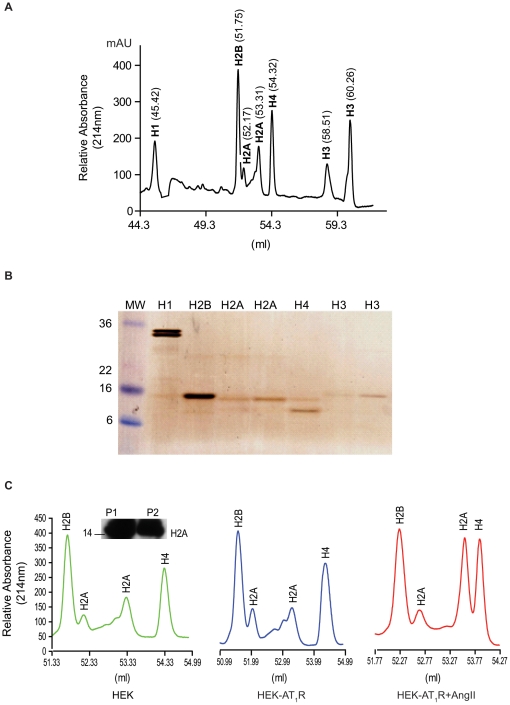
Profile of histones purified from HEK nuclear extract. (**A**) A typical reverse phase FPLC chromatogram of histones used for mass spectrometry analysis; (**B**) Silver stained SDS-PAGE of the FPLC fractions; each fraction was confirmed by matching the molecular weight in mass spec analysis; (**C**) comparision of FPLC peaks of histone H2A in the three experimental conditions; inset depicts western blot verifying both FPLC peaks as H2A.

We compared the histone profiles in HEK293 control cells, HEK cells that stably express AT_1_R (HEK-AT_1_R, 2.8 pmol/mg) [22], and the agonist-activated HEK-AT_1_R cells, grown and processed under identical conditions (Fig. 1). Total histones were isolated from the purified nuclei by acid extraction as described earlier [23], [24]. Mixed histones were separated by a C_18_ reverse-phase FPLC (Fig. 2A) and the fractions were characterized by SDS-PAGE analysis (Fig. 2B). Fractions of each FPLC peak were then pooled and analyzed directly by mass spectrometry on an ion-trap LTQ instrument. The reverse-phase FPLC, using a C_8_ column, was very efficient in resolving individual histones, yielding consistent and reproducible elution patterns for the three samples compared in this study (data not shown).

The FPLC chromatogram showed seven well resolved peaks (Fig. 2A), which after MS analysis and deconvolution of the multiple charged ion (8^+^ through 25^+^) series were identified as histone proteins H1, H2A, H2B, H3, and H4 (Table 1). The silver stained gel (Fig. 2B) corroborates these findings. The MS analysis yielded a range of masses for major histone in each FPLC peak (Table 1), providing evidence of molecular complexity in each histone type. In this study, we focused our analysis on the H2A fractions due to the fact that H2A consistently eluted in two distinct peaks in all samples. The two H2A peaks differed in the three samples reproducibly (n = 5) when compared to the H2B or H4 peaks, but displayed identical immuno-reactivity and mobility on the SDS-PAGE (Fig. 2B and C). The second H2A peak was broader in HEK-AT_1_R cells and the peak height increased substantially when cells were treated with AngII. In addition, previous reports have suggested potential modulation of H2A gene expression in mouse models of pressure overload cardiac hypertrophy [25]. We will embark on the analysis of other histone peaks following this report.

**Table 1:** Identification of histones in the FPLC peaks.

Number	FPLC-Peak (ml)	Mass range by mass spec	Histone assignment	Observed molecular weight (KDa)
1	45.42	22620-21805	H1	32–33
2	51.75	13815-13774	H2B	15
3	52.17	14046-13930	H2A-1	14
4	53.31	14046-13930	H2A-2	14
5	54.32	11742-11307	H4	11
6	58.51	15410-15187	H3-1	17
7	60.26	15645-15358	H3-2	17

Column one is retention ml for each peak. Column two is experimental mass range in each peak, column three shows the assignment of histone, and column four is relative molecular weight (KDa) based on mobility in SDS-PAGE shown in Fig. 2B.

### Complexity of histone H2A

The range of mass observed within H2A peaks led us to analyze the isoforms and PTM by LC/MS of intact H2A polypeptides. The molecular masses were determined for both H2A peaks. The relative abundances were calculated for molecular species that were consistently observed in the mass spectrum. A comparative analysis in the three samples provided an index of changes in H2A as the activation state of AT_1_R changed (Table 2).

**Table 2:** Isoform and PTM combinations observed in the H2A peaks from HEK cell lines.

		Mass (Da)			Relative Abundance (%)		
Assignment	13^+^ Charge state (m/z)	Deconvoluted	Calculated*	Δm (Da)	HEK	HEK-AT_1_R	HEK-AT_1_R +AngII
**First Peak**							
H2AA	1081.4	14047	14046.3	0.7	3	2	5
H2AA+1me	1083.4	10460	14046.3	13.7	2	5	3
H2AG	1079.5	14021	14018.3	2.7	21	22	15
H2AM	1084.3	14081	14083.4	−2.4	0	2	2
H2AO	1078.3	14008	14006.3	1.7	21	0	0
H2AQ	1071.3	13899	13899.2	−0.2	8	4	12
H2AQ+Ac/(3me)	1074.6	13945	13899.2	45.8	2	1	3
Q96QV6	1087.2	14141	14144.3	−3.3	1	4	4
**Second Peak**							
H2AC	1078.0	14002	14002.3	−0.3	21	30	22
H2AC+1me	1079.2	14014	14002.3	11.7	6	8	9
H2AC+1P	1084.1	14086	14002.3	83.7	4	2	2
H2AC+2me	1080.2	14029	14002.3	26.7	4	4	5
H2AC+3me/(Ac)	1081.1	14047	14002.3	44.7	6	10	11
H2AL	1091.9	14022	14016.3	5.7	1	1	2
H2AM	1084.3	14084	14083.4	0.6	0	8	6

Isoforms and PTMs observed in the H2A peaks from HEK cell lines. First and second histone H2A FPLC peaks were subjected to mass spectrometry analysis. Each row in the table represents a discrete H2A polypeptide species assigned based on the mass. Assignment was done by comparing measured masses to calculated molecular weights of histone isoforms based on primary sequences taken from the NCBI database and allowing for modifications. Column one is the assignment of H2A isoforms and modifications, column two contains m/z for each isoform and modification at 13+ charge state, columns three and four contain deconvoluted and calculated mass (from NCBI database) respectively; Δm, difference in mass between calculated and deconvoluted mass (Da). The last three columns contain the relative abundance (%) of the isoforms and their post translational modifications (p<0.05 in 3–5 determinations). The relative abundance is taken directly from Mass Scans.

*Mass values based on NCBI database.

The assignments were based on molecular weights that were calculated from the mass spectra that matched the predicted theoretical masses of different H2A subtypes (primarily human) from known amino acid sequences. Additionally, this assignment method allowed for post-translational modifications reported in the NCBI database and has previously been utilized [24]. It should also be noted here that the putative assignment of PTM in Table 2 is based purely on the consideration that mass increments of 14, and 28 Da are mono- and di-methylations, a mass difference of 42 Da corresponds to tri-methylation or an acetylation, and an 80 Da difference corresponds to phosphorylation [23], [24]. However, it is important to recognize that two isoforms differing by such nominal masses cannot be distinguished from post-translational modified isoforms based solely on mass differences. We did not, however, encounter isobaric species, which do not resolve. Hence, the assignment of H2A isoforms and PTM shown in Table 2 could be made unambiguously.

### Histone H2A isoforms

**Figure 3:**
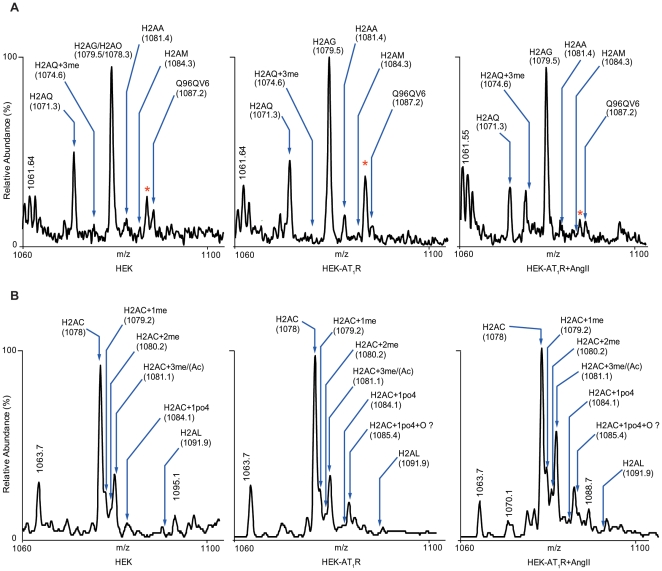
Mass spectrum of intact histone H2A from FPLC peaks showing resolution of isoforms and PTM. (**A**) The figure shows the spectrum expanded at 13+ charged state for all three (HEK, HEK-AT_1_R and HEK-AT_1_R+AngII) experimental conditions. The masses of the intact histone components were determined after deconvolution of the multiple charge ion series (not shown). This peak fraction contains multiple isoforms of H2A. This spectrum shows that H2A isoforms H2AG/H2AO (1079.5 m/z) did not appear in HEK-AT_1_R+AngII treated condition; the appearance of H2AM can also be seen in this peak (See Table 2). (**B**) Molecular masses of FPLC peak 2: intact histones from H2A peak determined after deconvolution of the multiply charged ion series, expanded at 13+ charged state for all three experimental conditions. In this peak fraction the major isoform is H2AC (1078 m/z) and its modifications. In this spectrum, phosphorylation of H2AC is reduced (H2AC+1P) (1084.1 m/z) in HEK-AT_1_R and HEK-AT_1_R+AngII conditions and an increase of H2AC monomethylation can be seen (See Table 2).

Figure 3 and Table 2 show an overview of the isoforms and PTM assigned for the two chromatographically distinct FPLC peaks. The mass spectrum of intact H2A histones allowed a first assignment of six potential isoforms in the first peak. Deconvoluted molecular masses at 13899, 14141, 14081, 14047, 14021, and 14008 in the first peak match well with the calculated masses of histone H2AQ (13899.2 Da), Q96QV6 (14144.3 Da), H2AM (14083.4 Da), H2AA (14046.3 Da), H2AG (14018.3 Da), and H2AO (14006.3 Da), respectively. The m/z 1078.3 (z = 13) species, characteristic of H2AO, was well resolved in the deconvoluted mass spectrum shown in Fig. 3A. This peak was distinct from the H2AG assigned (m/z 1079.5, z = 13) in HEK-AT_1_R and HEK-AT_1_R+AngII samples. Thus, the H2AO isoform appears to be a legitimate component of the H2A pool in HEK cells.

The mass spectrum of histones eluting in the second H2A peak shown in Fig. 2C, allowed assignment (see Table 2, Fig. 3B) of three isoforms H2AC (14002.3 Da), H2AL (14016.3 Da), and H2AM (14083.4 Da). In previous MS and MS/MS analyses in Jurkat cells, isoforms H2AO, H2AQ, and H2AZ were found in the first FPLC peak, and isoforms H2AA, H2AC, H2AE, H2AL, H2AG, and Q96KK5 were found in the second peak, without any overlap [24]. In our study, the H2AM isoform was present in both peaks and the composition of isoforms in the two peaks was different.

### H2A histone PTM

Potential PTM of histone H2A were apparent by screening the masses of the full-length H2A histone for known modification-specific mass increments. In H2A (first peak), the deconvoluted molecular mass at 14060 Da matched the 1-me form of H2AA (14046+13.7 Da). This form was observed in all samples (Table 2). The experimentally derived mass of 13945 Da did not directly match any human H2A isoforms in our list. Each sample showed this form. Calculated molecular weights from this ion gave the closest match to a possible acetylation (or tri-methylation) on the H2AQ isoform (MW: 13899+45.8 Da).

In the H2A (second peak), molecular masses 14014, 14029 and 14048 Da matched mono-, di-, and tri-methylated (or acetylated) forms of the histone H2AC (14002 Da) isoform (Table 2), which was consistently observed in all samples (Fig. 3B). Finally, mass 14086 Da matched the mono-phosphorylated molecular mass of H2AC variant (14002+83.7 Da). The m/z 1085.4 (z = +13) ion form was present in the mass spectra of AT_1_R expressing samples. A deconvoluted mass of this form corresponds to a PO_4_+O molecular species. The +16 modification (O) are commonly assigned to oxidation that can occur on both Met and Trp residues in proteins. Since we do not currently have sufficient information to assign this ion, the identity or significance of this species is not further characterized at this time.

### Activation states of AT_1_R influence the complexity of H2A

The relative heights of the two H2A peaks differed reproducibly in the three samples when compared to the H2B peak (Fig. 2C). The second H2A peak was broader in HEK-AT_1_R cells and the peak height increased substantially when treated with AngII. The changes observed in the FPLC profile were further substantiated by the mass spectrometry analysis (Table 2). The composition of isoforms in the H2A peak 1 (six isoforms) and peak 2 (three isoforms) changed in the HEK-AT_1_R and the AngII activated HEK-AT_1_R cells when compared to HEK cells. These samples contained substantially higher levels of isoforms H2AM and Q96V6, and completely lacked H2AO, a major isoform in HEK cells. In addition, the relative abundance of different isoforms was altered (see Table 2).

Dynamic changes in the post-translational modification of H2A histones are evident in our experiment(s). Nearly 16% of the H2AC variant was methylated in HEK control cells. In HEK-AT_1_R, this fraction increased slightly (21%), but the di- and tri-methyl forms significantly increased. This suggests either modification at additional sites on H2AC or further modification of mono-methylated residues. The total methyl-H2AC fraction increased to 25% upon AngII activation. Methyl-H2AA and acetyl-H2AQ (or me3) isoforms increased in the AngII activated HEK-AT_1_R cells. Together, these results indicate increased H2A methylation events in the presence of AT_1_R in cells. The phosphorylated H2AC (4%) present in HEK cells is substantially decreased, indicating H2AC dephosphorylation in the HEK-AT_1_R and AngII-activated HEK-AT_1_R cells. Taken together these findings suggest that the abundance of H2A PTM is altered significantly in the presence of AT_1_R. The methylation and dephosphorylation of the H2AC isoform could be novel AT_1_R-specific signaling events.

### Immunochemical analysis validates H2A isoforms and PTM

**Figure 4:**
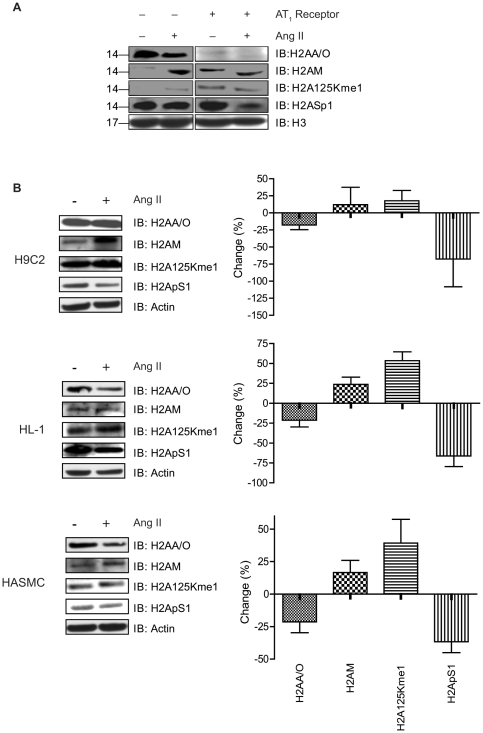
Immunochemical confirmation of isoforms and PTM of histone H2A. (**A**) Western blot analysis of nuclear extracts from HEK and HEK-AT_1_R cells treated with 1µM AngII for 1 hr using specified antibodies: H2AA/H2AO; H2A125Kme1; H2AM/H2A 25P hydroxyl; H4/H2A1Sp. The signal from H2AM/H2AP25hydroxyl antibody is indicative of H2AM, because we did not find hydroxyl-Pro in our MS analysis. The Ser-1p antibody signal is H2A-specific since we excluded H4 in the samples. Histone H3 Western blot was used as a loading control. (**B**) Western blot analysis of nuclear extracts from H9C2, HL-1 and HASMC cell lines treated with 1µM AngII for 5 hr using specified antibodies. Actin Western blot was used as a loading control. Band intensity values were obtained in three independent experiments. The right hand panels in 4B, shows mean ± SEM values for change compared to sample without AngII treatment.

To independently support the mass spectrometry results, we immunoblotted nuclear proteins using commercially available antibodies for H2A isoforms and PTM (see Fig. 4). The H2AA/H2AO isoform-specific antibody detected the H2A band in HEK cells, which decreased in HEK-AT_1_R and AngII activated HEK-AT_1_R cells. The H2AA/H2AO isoform-specific antibody signal substantiates MS data shown in Table 2 , (i.e., the absence of H2AO and the presence of a smaller amount of H2AA in the AT_1_R-expressing cells).

H2AM was detected by the H2AM/P25 hydroxyprolyl isoform-specific antibody in the HEK-AT_1_R and AngII activated HEK-AT_1_R samples. The H2A125Kme1 modification was detected both in HEK-AT_1_R and AngII activated HEK-AT_1_R cells at higher levels than in the control HEK cells. The H2A1Sp antibody gave a strong signal in the control cells, as predicted from the mass spectrometry data. This signal decreased substantially upon AngII activation in the HEK-AT_1_R cells.

We could not independently confirm the presence of methylated H2AA or acetylated H2AQ isofoms due to the fact that there are no commercially available antibodies specific for these modifications. Similarly, the immunochemical evidence for presence and exchange of isoforms H2AG, H2AQ, H2AC, and H2AL could not be obtained due to lack of commercially available antibodies. Thus, immunoblot analysis using four different H2A-directed antibodies validated the MS data which suggested that AT_1_R-induces changes in composition of histone H2A-isoforms and PTM. These four antibodies were utilized to further confirm the selectivity of AT_1_R in causing the changes in the composition of histone H2A-isoforms and PTM.

### Regulation of H2A isoforms and PTMs in response to endogenous AT_1_R activation

In the HEK293 cells the H2A histone changes in response to AngII treatment was variable (i.e., H2AM/P25hydroxyl signal in Fig. 4A), presumably due to low levels of AT_1_R as reported previously (http://www.tumor-gene.org/cgi-bin/GPCR/by_cell_line.cgi). To examine whether AT_1_R activation in untransfected cells regulates H2A isoforms and PTMs, we have used H9C2, HL-1 and HASMC muscle cell lines. Nuclear extracts from AngII activated cells were used for immunoblot analysis of H2A isoforms and PTMs (see Fig. 4B). The H2AA/O isoform decreased in all three cell types; the H2AM/P25 hydroxyprolyl was increased. The level of H2A125Kme1 modification was higher and the H2A1Sp decreased substantially in all three cell types (see Fig. 4B). Losartan blocked these AngII-induced changes in all three cell lines (data not shown).

### The activation states of AT_1_R alter the dynamics of histone H2A isoforms

**Figure 5:**
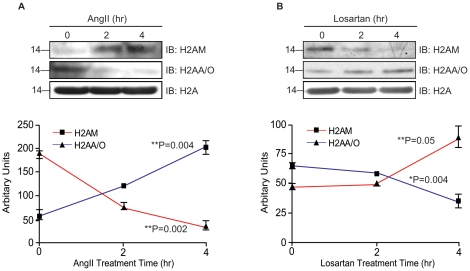
Time course analysis of ligand-dependent exchange of H2AA/O and H2AM isoforms. The H2A isoforms were immunoblotted using isoforms-specific antibodies, H2AA/O and H2AM/H2A 25P hydroxyl after treatment of HEK-AT_1_R with AngII (1µM) or losartan (1µM). Each data point shown represents mean ± SEM of 3–5 independent experiments. Significance values shown are for comparison between 0 and 4 hr time points, obtained by one-way ANOVA followed by unpaired Student *t*-test. *P*-values less than 0.05 are considered statistically significant.

The states of AT_1_R induced by AngII and losartan dynamically regulate isoforms H2AA/O and H2AM. AngII-stimulated an increase of H2AM and a disappearance of the H2AA/O isoform in 2–4 hr time-kinetics (see Fig. 5A). Treatment of HEK-AT_1_R cells with losartan showed decrease of H2AM by 4 hr (Fig. 5B); whereas, the H2AA/O isoforms showed an increase as anticipated. Losartan is an inverse agonist of AT_1_R. Hence, the result of the losartan treatment confirms direct involvement of AT_1_R in the exchange of these two H2A isoforms.

### Ligand-dependent alteration of H2A-PTMs and association with functional molecules

**Figure 6:**
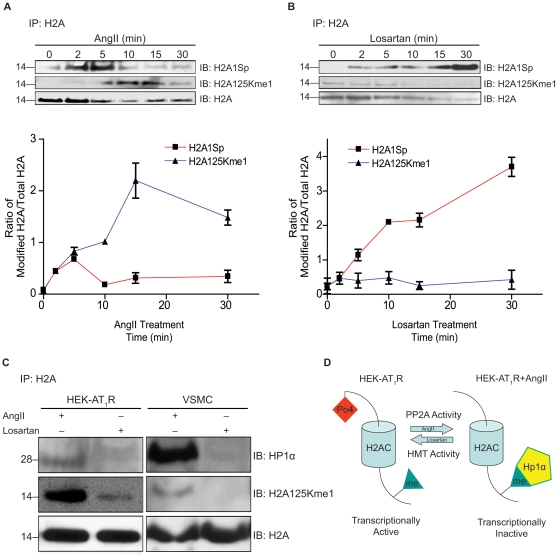
Time course analysis of ligand-dependent change of H2A-PTMs. Dephosphorylation of H2A detected by H2A1Sp antibody and monomethylation of H2A detected by the H2A125Kme1 antibody. (**A**) Time course of H2A1Sp and H2A125Kme1 signal modulation in HEK-AT_1_R treated with AngII (1µM); (**B**) HEK-AT_1_R cells treated with losartan (1µM). (**C**) H2A125Kme1 interaction with HP1α in HEK-AT_1_R and HASMC treated with AngII (1µM) or losartan (1µM) for 1 hr. Nuclear extracts of various samples were immunoprecipitated using pan-H2A antibody followed by immunoblotting for H2A125Kme1 and HP1α in the immunoprecipitate, (**D**) Schematic representation of functional consequences of two H2A-PTMs affected by activation states of the AT_1_R.

The dynamic regulation of H2A-PTM is rapid when compared to the exchange of isoforms. Phosphorylation of H2AC1Ser increased transiently up to 5 min after AngII treatment, and was followed by a decrease to a steady-state level in HEK-AT_1_R cells (Fig. 6A). When HEK-AT_1_R cells were treated with losartan, this process displayed an opposite trend, reaching a higher steady-state level of H2AC1Ser phosphorylation than untreated HEK-AT_1_R (Fig. 6B). H2A methylation, which was monitored by an H2A125Kme1 antibody, increased upon AngII treatment (Fig. 6A) and was inhibited by losartan treatment (Fig. 6B).

To decipher possible functions mediated by these two PTMs, we examined interacting proteins. H2A1Sp was associated with a catalytic subunit of protein phosphatase 2A. We then measured the phosphatase activity associated with the H2A1Sp immunoprecipitate. Upon AT_1_R activation the associated phosphatase activity decreased, which correlates with the decrease of H2A1Sp. When AT_1_R signaling was blocked by treatment with losartan, the phosphatase activity associated with H2A increased. The histone methyl transferase (HMTase) activity associated with H2A increased upon AT_1_R activation and the association decreased when the AT_1_R signaling was blocked with losartan. Together, these results indicated that H2A1Sp and H2A125Kme1 modifications promote the association of phosphatase and HMTase with the chromatin.

We immunoprecipitated H2A and probed for H2A125Kme1 and the associated heterochromatin marker protein HP1α in HEK-AT_1_R and vascular smooth muscle cells. As shown in Fig. 6C, when H2A125Kme1 was present, the association of HP1α was significantly higher. When treated with losartan, the association with HP1α is reduced, thus suggesting that the HP1α interaction is facilitated by the H2A125Kme1 modification that is induced by AT_1_R activation. In previous studies Hirota *et al.*, 2005, found that the phosphorylation of histone H3 at Ser-10 negatively regulated the binding of HP1α to the adjacent methylated Lys-9 of histone H3. Based on their study of histone H3 modifications, these authors proposed the binary switch hypothesis [26]. We propose that dephosphorylation of the N-terminal H2A1Sp may induce H2A125Kme1 modification at the C-terminus. This modification when induced upon AT_1_R activation promotes hetrochronatin formation, consequently silencing gene transcription (Fig. 6D). The changes in histone H2A-PTMs when induced upon AT_1_R activation are functionally significant in altering the expression of genes. It is of great interest to note that pharmacological inhibition of AT_1_R produces a change in chromatin histone composition with functional significance.

## Discussion

In this study we present evidence of changes in histones in HEK293 cells that occur upon *de novo* expression of AT_1_R as well as ligand activation of AT_1_R. These AT_1_R-dependent changes are reversible upon inhibition of AT_1_R signaling by losartan. Alteration of chromatin structure in response to change in the activation-state of AT_1_R or any other GPCR is not documented. Modulation of histones by GPCR is most likely a *cis*-acting epigenetic chromatin regulatory mechanism. The H2A isoforms and PTM could directly affect chromatin structure and function of chromatin which is critical for the modulation of global gene expression. Finding a link between AT_1_R activation/inhibition and histone complexity in chromatin is imperative for a better understanding of molecular mechanisms of phenotypic modulation by AngII in various cell types. Conversely, reversal of AT_1_R-induced histone changes in cardiac, vascular and renal cells may be an essential but uncharted mechanism during therapeutic intervention with AT_1_R blockers. Therefore, a complete description of specific chromatin changes induced by AT_1_R is desirable. However, because of the enormity of changes hidden in the profile of each of the histones (Table 1), we restricted our study to H2A histones. Our reasons for selecting H2A are: (i) limited information on isoforms and PTM for H2A relative to core histones H3 and H4; and (ii) a previous study, suggesting that the H2AZ isoform is differentially regulated during pressure-induced cardiac hypertrophy [25]. Our current study is a novel and significant effort towards our long-term goal to pursue comprehensive analyses of histone H2A isoforms at the protein level in health and disease.

**Figure 7:**
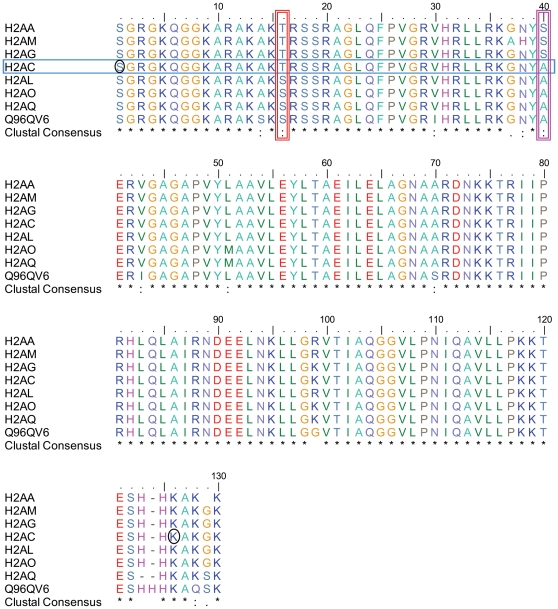
Sequence comparison of human H2A isoforms. The vertical boxes highlight the 16^th^ and 40^th^ amino acid residue position in the eight H2A variant sequences. The horizontal box shows the unique feature of H2AC, which has a threonine at the 16^th^ position, but an alanine at the 40^th^ position. We speculate that interactions of Thr-16 and Ala-40 in the H2AC variant may play a role in AngII-responsive Ser-1 dephosphorylation and Lys-125 methylation, the residues are shown in black circles.

A key question we explored in this study is the influence of the *de novo* expression of AT_1_R on the composition of core nucleosome components in HEK293 cells. We examined changes in nucleosome composition in cells expressing endogenous levels of AT_1_R (Fig. 4B). The FPLC purification resolved the histones H1, H2A, H2B, H3, and H4 into individual peaks (Fig. 1) and enabled MS analysis of intact histones to document inherent molecular heterogeneity (Table 1). Each H2A peak contained a mixture of several isoforms (Fig. 2, Table 2). The isoforms H2AA, H2AG, H2AM, H2AO, H2AQ, and Q96QV6 were identified in the first FPLC peak; whereas, H2AC, H2AL, and H2AM, were found in the second FPLC peak. These isoforms are products of various genes that encode slightly different primary sequences shown in Fig. 7 [26], [27]. The human genome encodes four replacement H2A histones; H2AZ, macroH2A, H2A-Bbd, and H2AX. We did not find evidence of replacement histones in the FPLC peaks analyzed in any of the samples [28], [29]. Histone H2AZ, reported to be important in cardiac hypertrophy [28], [29], was not detected in any of the samples, suggesting that H2AZ is not responsive to AngII.

Although the possibility of not recovering all minor isoforms and rare modifications cannot be ruled out in our analysis, the MS data already suggests that the AT_1_R activation/inhibition causes fluctuation of several interesting and perhaps unique isoforms (i.e., Q96QV6, H2Q, H2AAme1, H2AQAc and H2AL) and PTM (Table 2), which must alter the composition of the nucleosome. The exchange of H2AA/O and H2AM isoforms is clearly an AT_1_R-specific signaling event since AngII treatment increased H2AM and decreased H2AA/O. Losartan treatment increased H2AA/O, as we anticipated from the inhibition of the constitutive activity of AT_1_R by losartan (Figs. 4 and 5B). The PTMs of H2AC isoform assessed by mass spectrum (Fig. 3A and B) and biochemical analysis (Figs. 4 and 6) also indicates AT_1_R-specific modulation. Unavailability of reliable isoform-specific antibodies limited extensive analysis of all isoforms in this study. The AT_1_R-induced H2AA/O exchange with H2AM documented here is suggestive of a genome-wide pattern of exchange. AngII-induced histone changes in cardiac and smooth muscle cell lines (Fig. 4B) demonstrates that the histone changes may play a role *in vivo* in directly regulating the long-term response of genes or induce changes in phenotype of these cells. For instance, minor histone isoforms are known to be placed at strategic locations in the genome, such as gene regulatory regions [30], [31], [32]. This would provide a mechanism for regulating genes through variegation of chromatin domains and alter higher order chromatin structure. The differences in the sequence of histone isoforms affects chromatin function by providing binding sites to other histone isoforms, modifying enzymes or transcription factors and thus may alter nucleosome stability [26].

Reciprocal modulation of H2AC PTM, 1Serp and 125Kme1, by AT_1_R activation/inhibition is an important discovery. Of all H2A isoforms, H2AC is unique in containing a threonine at position 16 and alanine at position 40 (see Fig. 6). These substitutions together could produce a relatively less compact nucleosome core. As a result, the H2AC1Ser at the N-terminus or H2AC125K near the C-terminus may become more sensitive to dephosphorylation and methylation, respectively, than other H2A isoforms upon AT_1_R activation. Previous studies indicate that H2A PTM affect gene expression [33], [34], [35], such as transcription by GAL4 and MSK1 [35] and mitotic chromosome condensation [36]. Several PTM described in the literature for H2A histones [24], [37], [38] were not found in our model, which is a AT_1_R over expressing human cell line. For example, human histone H2A modifications already reported include acetylation on Lys-5 and Lys-9, ubiquitination on Lys-119 in the carboxyl-terminal region [39], [40], and phosphorylation of H2AX Ser-139. H2A125Kme1 has also been shown in cancer cells [41], [42].

In general, core histone PTMs regulate one another and can influence the occurrence of subsequent modifications either on the same or an adjacent nucleosome. Therefore, H2AC1Sp dephosphorylation coupled to methylation of H2AC125K affected by the activation/inhibition of AT_1_R is consistent with the idea of a simple redundant ‘histone code’ as seen in several other regulatory systems [43], [44], [45], [46]. The histone code is deciphered by binding of effectors that further modify the ‘histone code’ and modulate specific gene activity [43], [44], [45]. Therefore, the association of PP2A and HMTase activities with H2AC regulated by AT_1_R activation/inhibition is significant. Dephosphorylation of H2AC1Sp could open the C-terminal tail domain of H2AC for association with HMTase leading to methylation of H2AC125K which facilitates interaction with the heterochromatin protein HP1α. This interaction is likely to be involved in directing the HP1α-bound nucleosomes to heterochromatic regions that are known to be enriched in unphosphorylated H2A. Dephosphorylation of nucleosome core histones could promote methyl-Lys-bound HP1α to self-associate within a region of chromatin [47], [48], [49]. A recent study [50] has shown that the HP1α can also associate with other HP1 isoforms which in turn can bind to the linker histone H1b. Therefore, the modification status of histone H2AC could change the balance between transcriptionally active and inactive chromatin regions. The H2A phosphorylation upon losartan would also influence the chromatin structure because it would lead to a decreased affinity for HP1α. Thus, the model shown in Fig. 6D provides a mechanism by which AT_1_R signaling leads to HP1α mediated regulation of chromatin activity.

Exchange of H2AA/O isoforms with H2AM (Fig. 5) appears to be a slower process than PTM changes. The consequence of isoform exchange may last longer and changing PTM may be important to moderate acute response. Altering chromatin response to AT_1_R *in vivo* may utilize both mechanisms, but the emphasis on altering PTM or isoforms may differ under chronic and acute conditions. Long-term treatment with AT_1_R blockers may reset gene expression by altering the composition of isoforms and PTM. Histone changes of promoter-proximal nucleosome provide the syntax (or context) that correlates with transcriptional activity *in vivo*. The ability of RNA polymerase II to cross the first nucleosome it encounters is a major, and general, control point for gene expression. Nucleosomes positioned after the TATA box form a strong blockade to RNA polymerase and the exchange of H2A/H2B dimers is a critical step that reduces the barrier for nucleosome traversal of RNA polymerase at transcribed genes [51]. How AT_1_R might regulate this process through its influence on H2A would be important to discern. Hence, considering AT_1_R-induced chromatin changes as part of the mechanism of global gene expression is critical. The study of AT_1_R-sensitive PTM and isoforms controlling expression of specific genes in cardiac hypertrophy and heart failure are of great importance.

**Figure 8:**
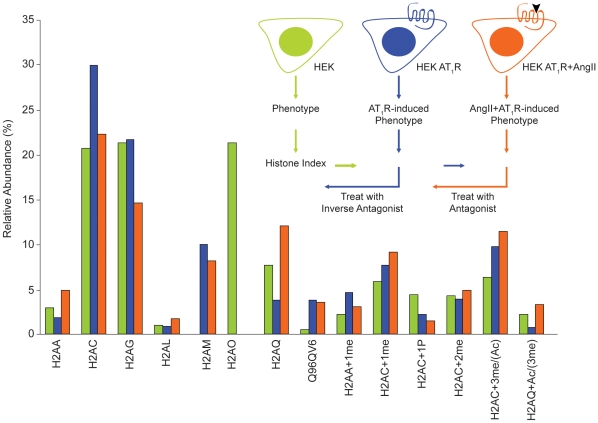
Model for modulation of H2A histone index in AT_1_R signaling. The bar graph summarizes the H2A index deduced in this study. The scheme shows reversible changes in the histone code influenced by activation states of the GPCR, AT_1_R.

Figure 8 summarizes our results. The H2A histone index is responsive to the functional states of the AT_1_R, a GPCR. AT_1_R initiated swapping of histone isoforms and modification of histones in the nucleosome is a regulatory cryptogram for deciphering the encoded genomic information in the context of regulating the gene expression. We suggest that histone replacement and modifications associated with activation states of GPCRs may play an important role in the progression of disease. GPCRs activate rapid transmission of signals in mediating acute and chronic adaptive responses of cells to a host of ligands. GPCRs also amplify cellular response by activating transcription factors and components of the transcription machinery. However, the dynamic regulation of the structure of chromatin has largely been neglected as a relevant target of GPCR signaling pathways. In essence our work eludes to the regulation of the histone code by a GPCR [52], which accounts for many chromatin functions, including transcriptional regulation through *cis*-acting (local and global chromatin compaction), as well as transacting (recruitment of transcriptional activators and inhibitors) mechanisms of epigenetics [53]. GPCRs are the known targets for >50% of approved drugs and intended targets of novel drug development efforts [2], [4], [12], [54], [55], [56]. Drug therapy targeting GPCRs may reverse the changes in the histone code. The classical antagonists and inverse agonists may exert significantly different influences on the reversal of the histone code (see Fig. 8).

In summary, we presented the analysis of H2A histone isoforms and PTM in HEK cell lines each tuned to a distinct activation state of a GPCR, the AT_1_R for the peptide hormone AngII. This is the first reported analysis of H2A histone remodeling that occurs in chromatin upon expression and hormone-stimulation of a GPCR. Overlapping and distinctive changes seen in HEK-AT_1_R and HEK-AT_1_R+AngII cells might indicate a scenario one might anticipate in chronic and acute influence of AT_1_R. The histone index may influence the physiological state of cells modifying cellular phenotypes in the process of disease, as well as provide new targets for drug therapy.

## Materials and Methods

### Preparation of nuclei from cell lines and extraction of histones

Human embryonic kidney cell line (HEK293) purchased from American Type Culture Collection (ATCC, Manassas, VA). (HEK293) stably harboring pCDNA3 empty vector, HEK293 harboring pCDNA-AT_1_R vector stably expressing 2.8 pmol/mg AT_1_R (HEK-AT_1_R) [22], HEK-AT_1_R line treated with 1µM AngII for 1 hr (HEK-AT_1_R+AngII) were cultured in DMEM medium containing 10% FCS, 100 IU penicillin/streptomycin, and 600µg/ml G418. The H9C2 cell line originally derived from embryonic rat heart was purchased from ATCC, (catalog # CRL-1446). HL-1 cardiac myocytes were cultured in Claycomb medium containing 10% FCS [57] and serum starved before treatment with 1µM AngII for 5 hr. The human aortic smooth muscle cells (HASMC) were a gift (A. Majors) [58]. The HASMC was cultured in DMEM medium containing 10% FCS, 100 IU penicillin/streptomycin, and serum starved cell lines treated with 1µM AngII for 5 hr. Cells were washed twice with PBS, the cell pellet was suspended in lysis buffer (250 mM sucrose, 50 mM Tris-HCl, pH 7.5, 25 mM KCl, 5 mM MgCl_2_, 1% protease inhibitor (Sigma), 50 mM NaHSO_3_, 45 mM sodium butyrate, 10 mM β-mercaptoethanol, 0.2% Triton X-100) and centrifuged at 800× *g* for 15 min to obtain nuclei. For preparing histones, nuclei were extracted in 6 volumes of 0.2 M H_2_SO_4_ (16 hr at 4°C). After centrifugation at 16,100× *g*, supernatants were precipitated with trichloroacetic acid (25% final concentration). The pellet was washed with 1% HCl in acetone, followed by 100% acetone, and subsequently dissolved in β-mercaptoethanol (0.1%) in water. This protocol is modified from that described in references [23], [24].

### FPLC separation of histones

Bulk histones obtained by acid extraction of HEK cells were fractionated on a C8 column (220×4.6 mm Aquapore RP-300, Perkin-Elmer) using a linear ascending gradient of 0–100% solvent B (solvent A, 5% acetonitrile, 0.1% TFA; solvent B, 90% acetonitrile, 0.1% TFA) for >75 min at 1.0 mL/min on an AKTA FPLC system (Amersam Bioscience). The elution profile was monitored at 214 nm, and 0.5 mL fractions were collected. Fractions from each peak were pooled, dried under a vacuum, and stored at −80°C. Prior to MS analysis an aliquot from each fraction was resolved on a 4–12% SDS-PAGE gel to assess the purity of the fractionated histones [24].

### Mass spectrometry

Preparative FPLC fractions containing individual histones, as detected by UV and MS chromatograms, were pooled. Bradford assay gave a tentative concentration of ≈0.02µg/µL for fraction 2 and ≈0.08µg/µL for fraction 3. For MS analysis, ≈50µL of each sample was dried in a SpeedVac and then redesolved in ≈5µL of 1.0% aqueous formic acid.

The LC-MS system is a Finnigan LTQ-linear ion trap mass spectrometer system equipped with a nanoelectrospray source and interfaced to a self-packed 9 cm×75µm inner-diameter Phenomenex Jupiter C18 reversed-phase capillary chromatography column. The sample (0.5 to 2µL) was injected, based on the approximate concentration of protein as assessed by the chromatographic peak heights. Proteins were then eluted from the column by an acetonitrile/0.1% formic acid gradient (0–100%) at a flow rate of 0.2µL/min. The nanoelectrospray ion source was operated at a spray voltage of 2.5 kV and capillary temperature of 160°C. Profile mass scans were acquired in the positive ion mode within the range of 300–1800 Da. The mass accuracy of the instrument for intact proteins was set to be 100 ppm in the analysis. The molecular masses of the histones were determined by deconvoluting the multiple charged ion series, which typically ran from 8^+^ through 25^+^ (Bioworks Software, Thermo Finnigan). Assignment of the correct histone isoforms was performed by comparing the measured masses to calculated masses of histone sequences taken from the NCBI database (primarily human) (see Table 2).

### Immunoblotting and immunoprecipitation

Total nuclear extract and immunoprecipitated (with anti-H2A and anti-H2A1Sp) samples were separated by gel electrophoresis on an 18% SDS–PAGE gel before being transferred to the PVDF membrane (BioRad) as previously described [59]. Blots were probed with a 1∶5000 dilution of anti-H2A polyclonal antisera (Upstate); 1∶1000 dilution of anti-H2AA/H2AO (Novus Biologicals); and a 1∶250 dilution of anti-H2A hydroxyl P26/H2AM (Abcam) in 5% non-fat milk, PBS, 0.1% Tween-20, followed by incubation with a horseradish peroxidase conjugated goat anti-rabbit, 1∶250 dilution of anti-H2A mono-methyl K125 (Abcam) anti-mouse in 5% non-fat milk, PBS, 0.1% Tween-20, followed by incubation with anti-mouse secondary antibody, 1∶5000 dilution of anti-H2A/H4 phospho Ser1 (Active Motif) anti-rabbit in 5% non-fat milk, PBS, 0.1% Tween-20, followed by incubation with anti-mouse secondary antibody. Detection was performed using ECL chemiluminescence substrate (Amersham Pharmacia).

### Histone methyltransferase assay

We performed the assays as described earlier [59] using an HMT Assay Reagent Kit (Upstate). H2A-associated methyltransferase assays were completed by incubating 50µg of histone H2A immunoprecipitate with *S*-adenosyl-[methyl-^3^H]-l-methionine (25µCi/mL) and/or *S*-adenosylmethionine (New England BioLabs) as the methyl donor. Reactions were performed at 30°C for 80 min. To stop the reaction, acetic acid was added to a final concentration of 10% (v/v).

### Phosphatase activity assay

PP2A activity was determined using a PP2A Immunoprecipitation Phosphatase Assay Kit (Upstate). After the indicated treatments, cells were washed twice in cold phosphate-buffered saline. Nuclear lysates containing 200µg of protein were incubated with 4µg of anti- H2A1Sp and 40µL of protein A-agarose slurry for 2 hr at 4°C with constant rocking. The immunoprecipitates were washed three times in tris-buffered saline one time with Ser/Thr phosphatase assay buffer (50 mm Tris-HCl, pH 7.0, 100µm CaCl_2_), and resuspended in 20µL of the assay buffer. The reaction was initiated by the addition of 60µL of phosphopeptide substrate (750µm) (KRpTIRR). Following incubation for 10 min at 30°C in a shaking incubator, the reaction mixture was briefly centrifuged and the supernatant transferred to a 96-well microtiter plate. The reaction was terminated by the addition of malachite green phosphate detection solution for 10–15 min at room temperature, and free phosphate was quantified by measuring the absorbance of the mixture at 650 nm using a microplate reader.
